# Clinical evaluation of bacterial DNA using an improved droplet digital PCR for spontaneous bacterial peritonitis diagnosis

**DOI:** 10.3389/fcimb.2022.876495

**Published:** 2022-08-18

**Authors:** Hao-Xin Wu, Wei Hou, Wei Zhang, Zheng Wang, Shan Guo, De-Xi Chen, Zhen Li, Feili Wei, Zhongjie Hu

**Affiliations:** ^1^ Beijing YouAn Hospital, Capital Medical University, Beijing, China; ^2^ Beijing Institute of Hepatology, Beijing YouAn Hospital, Capital Medical University, Beijing, China

**Keywords:** Peritonitis, BactDNA, diagnosis, bacterascites, viable bacteria

## Abstract

**Objective:**

Bacterial DNA (bactDNA) detection can be used to quickly identify pathogenic bacteria and has been studied on ascitic fluid. We aimed to retrospectively analyze the diagnostic value and applicational prospect of the bactDNA load in spontaneous bacterial peritonitis (SBP).

**Method:**

We extracted viable bactDNA from ascitic samples of 250 patients with decompensated cirrhosis collected from October 2019 to April 2021 and detected the bactDNA by droplet digital polymerase chain reaction (ddPCR). We used ascitic samples of a baseline cohort of 191 patients to establish diagnostic thresholds for SBP and analyze the patients’ diagnostic performance based on ascites polymorphonuclear (PMN) and clinical manifestation. We performed bactDNA quantification analysis on 13 patients with a PMN less than 250 cells/mm^3^ but with clinical symptoms. The dynamic changes of the bactDNA load from eight patients (before, during, and after SBP) were analyzed.

**Results:**

After the removal of free DNA, the bactDNA detected by ddPCR was generally decreased (1.75 vs. 1.5 log copies/µl, P < 0.001). Compared with the traditional culture and PMN count in the SBP diagnosis, the bactDNA showed that the ddPCR sensitivity was 80.5%, specificity was 95.3%, positive predictive value was 82.5%, and negative predictive value was 94.7%, based on clinical composite criteria. In patients with a PMN less than 250 cells/mm^3^, the bactDNA load of 13 patients with symptoms was significantly higher than those without symptoms (2.7 vs. 1.7 log copies/µl, P < 0.001). The bactDNA in eight patients had SBP that decreased by 1.6 log copies/µl after 48 h of antibiotic treatment and by 1.0 log copies/µl after 3 days of continued treatment.

**Conclusion:**

BactDNA detection can be used to further enhance the diagnostic efficiency of SBP. Therefore, the application of ddPCR assay not only can be used to discriminate and quantify bacteria but also can be used in the clinical assessment for antibiotics treatment.

## Introduction

Spontaneous bacterial peritonitis (SBP) is an infectious disease caused by pathogenic microorganisms that invade the abdominal cavity and cause obvious damage (European Association for the Study of the Liver., 2018). In people with end-stage liver diseases, the incidence rate of SBP has been shown to reach 40% to 70% (Aithal et al., 2021).. Currently, SBP is defined as an ascites polymorphonuclear (PMN) count greater than 250 cells/mm^3^ (European Association for the Study of the Liver., 2018; Aithal et al., 2021; Biggins et al., 2021). However, approximately 60% to 80% of patients with a PMN less than 250 cells/mm^3^ have signs and symptoms (Oey et al., 2018; Song and Jiang, 2016); of those patients, 38% develop SBP (Li et al., 2020). However, empirical antibiotic therapy that is based on the patient’s clinical symptoms and PMN can lead to the excessive application of antibiotics and the occurrence of multi-drug resistant organisms (Piano et al., 2019; Fernández et al., 2019). Current traditional culture for SBP has insufficient sensitivity to detect samples with bacteria (European Association for the Study of the Liver., 2018), especially those with low bacterial loads. Therefore, it is urgent for researchers and clinicians to introduce more accurate and rapid etiological diagnosis methods.

Recently, ascites bacterial DNA (bactDNA) detection has been expected to replace the general bacterial culture of ascites in the identification of infectious pathogens (Enomoto et al., 2018; Aithal et al., 2021). Droplet digital polymerase chain reaction (ddPCR) is a novel absolute quantitative molecular detection technology that has been developed in recent years with advantages of high sensitivity, simplicity, fastness, and operation without relying on the standard curve (Hussain et al., 2016; Cho et al., 2020). The ddPCR technology produces about 20,000 droplets and enriches target DNA by reducing competition with high-copy templates. After PCR amplification in each droplet, the Poisson algorithm is used to determine the concentration of target DNA from positive and negative droplets. Studies have suggested that the ddPCR technology is able to detect very low amounts of pathogen DNA within 4 h and had been applied in the diagnosis of bloodstream and tuberculosis infections (Cho et al., 2020; Wouters et al., 2020). Those findings provide direction for the application of ddPCR in clinical diagnosis of bacterial infection.

Nevertheless, some previous studies (Zapater et al., 2008; Bruns et al., 2016; Alvarez-silva et al., 2019) have shown that there was no strong correlation between the presence of bactDNA in ascites and SBP; as a result, the diagnosis of SBP by bactDNA has not been readily applied in clinical practice. The possible reasons are as follows: 1) bacterial load plays a very important role in the development of SBP, not just the presence of bactDNA (Bernardi et al., 2020); 2) the large volume of ascites that often occurs in patients with cirrhosis may dilute bacterial products (Akalin et al., 1983); and 3) accumulated evidence indicates (Guarner et al., 2006) that bacteria may translocate either in a viable or non-viable form, and the migration of nucleic acid DNA may potentially obscure any correlation between microbes and clinical parameters.

Therefore, the purpose of our study was to assess the amounts of viable bactDNA in ascites for the diagnostic accuracy of SBP, using an optimized method of ddPCR.

## Methods

### Study design and population

**Figure 1:**
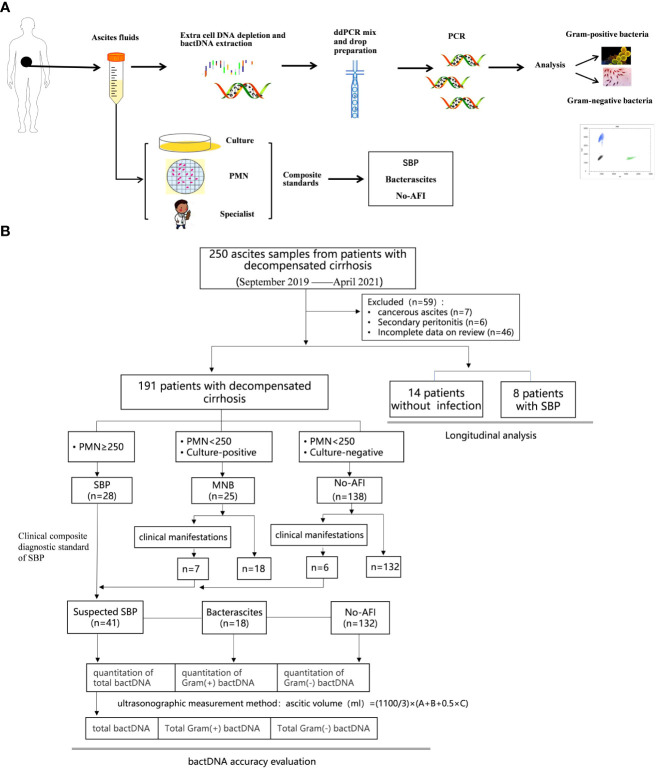
Study workflow and sample distribution. **(A)** Schematic of ddPCR ascites analysis workflow. A total of 1.5–2 h were needed for nucleic acid extraction and 2 h for the preparation, amplification, and analysis in ddPCR quantitation detection. **(B)** Overall flow of patients in the study showing patient recruitment and subsequent selection for bactDNA quantitation analysis. ddPCR, droplet digital PCR; PMN, polymorphonuclear; SBP, spontaneous bacterial peritonitis; MNB, monomicrobial non-neutrocytic bacterascites; AFI, ascites fluid infection. Gram(+), Gram-positive; Gram(−), Gram-negative.

This study was approved by the ethical committee of Beijing YouAn Hospital, Capital Medical University. The study design was composed of laboratory and clinical studies (**Figure 1**). Clinical data of the patients enrolled in the study were obtained by medical record review and were analyzed according to clinical course to determine the likelihood of an infection. A total of 250 patients with decompensated cirrhosis and ascites from the Liver Disease Center, Beijing YouAn Hospital, Capital Medical University, between September 2019 and April 2021 were retrospectively collected in the study. Cancerous ascites, secondary peritonitis, and incomplete clinical data were excluded. Ascitic samples of the first baseline cohort from 191 patients were obtained on admission. The SBP diagnosis is based on PMN (2021 practice guideline) (Biggins et al., 2021) and a clinical composite diagnosis (2017 Chinese guidelines) (Chinese Society of Hepatology, Chinese Medical Association, 2017) that incorporated 1) clinical signs or symptoms, 2) laboratory test abnormalities, and 3) adjudication independently by an infectious disease specialist (CLH) and two liver disease experts (YH and WH). Patients were initially classified as either SBP (PMN > 250/mm^3^), monomicrobial non-neutrocytic bacterascites (PMN < 250/mm^3^ with positive ascites culture), or ascites without infection (no-AFI; PMN < 250/mm^3^ with negative ascites culture) (European Association for the Study of the Liver., 2018; Biggins et al., 2021). Patients who had overt clinical symptoms but with a PMN count less than 250/mm^3^ were further classified as suspected SBP, bacterascites, and no-AFI (**Figure 1B**) (Rimola et al., 2000; Campillo et al., 2002; Gu et al., 2021). Subsequently, we selectively enrolled 14 patients without infection to observe an association between a higher baseline bactDNA load and SBP development and eight patients with SBP to analyze the dynamic changes of bactDNA load (before, during, and after antibiotics). The diagnostic standard and the inclusion and exclusion criteria are detailed in the Supplementary Data.

To evaluate and eliminate the influence of ascitic volume on bacterial quantity, we adopted the ultrasonic three-point method from Hirooka et al. (2018); this method is used to calculate the total amount of ascites: total amount (ml) = (1,100/3) × (A + B + 0.5 × C). In this way, the absolute total amount of bacteria could be determined by the total ascitic volume [(ml) × bacterial ddPCR (copies/µl)].

### ddPCR methods

#### Primes and probes

The representative 20 bacteria sequences of primers and probes were adopted and revised from the previous article and synthesized by Sangon Biotech (Shanghai) Co., Ltd (Wouters et al., 2020). The reaction conditions were optimized and screened according to the ddPCR requirements. Then, the performance of primers and probes was verified, which included sensitivity, specificity, linearity, and repeatability.

#### Sample processing

To deplete the extracellular DNA from the ascitic samples, 1-ml samples were centrifuged at 4°C with 13,000r for 10 min. After discarding the 760-µl supernatant, we added 40.5-µl mixture of buffer and benzonase endonuclease and incubated this mixture at 37°C for 15 min. Next, we added 20 µl of protease K and incubated it at 56°C for 20 min to inactivate the benzonase.

#### DNA extraction

Approximately 400 µl of 2× DNA/RNA shield (zymo R1200-125) was added into each of the pre-treatment samples. After mixing, the samples were homogenized using a program of 4°C at 60 Hz for 120 s, stopped for 20 s, repeated four times in Tissuelyser (Servicebio, Wuhan, China), and then centrifuged at 10,000r for 2 min. We then individually added 400 µl of DNA/RNA analysis buffer, prep buffer, and wash buffer for repeated DNA washing. Finally, 50 µl of DNase/RNase-free water was added to collect target DNA and stored at −80°C until tested.

#### Droplet preparation and detection

The ddPCR was performed with the TargetingOne Digital PCR System (TargetingOne, Beijing, China). The master mix for ddPCR included 1× ddPCR supermix for probes, forward and reverse primers at 400 nmol/L, and GRAM+/GRAM− probes at 200 nmol/L; 1-µl sample of DNA and DNase/RNase Free water were mixed together, and the final volume was 30 µl for each well. The droplet was generated according to the manufacturers’ protocols.

PCR amplification was performed with the following conditions: 95°C for 10 min followed by 40 cycles of denaturation at 95°C for 10 min; annealing and extension was at 60°C for 1 min. The strip tubes were stored at 4°C until the droplets were analyzed with a TargetingOne chip reader and TargetingOne ddPCR Analyzer 1.0. The threshold between positive and negative droplet populations was manually set using per-plate positive and no-template controls as a guide.

### Statistical analysis

The data are presented as a mean ± standard deviation or median with a range. We used the Wilcoxon–Mann–Whitney test for between-group comparisons or the Kruskal-Wallis test with *post hoc* tests for continuous data. The continuous variables were dichotomized according to the maximum approximate index in the receiver operating characteristic (ROC). The statistical analyses were performed using SPSS software, version 24 (IBM, Armonk, NY), and Prism 8 (GraphPad, La Jolla, CA). In the two-sided test, a P-value of <0.05 was considered statistically significant.

## Results

### Patients’ characteristics

Two hundred and fifty patients were enrolled in this study. We excluded seven patients with cancerous ascites, six patients with secondary peritonitis, and 46 patients who had incomplete data. One hundred and ninety-one patients were enrolled, and their ascitic samples were collected. Among them, 155 patients were men (81.2%), with an average age of 58.1 ± 9.1 years. Liver cirrhosis was caused by alcoholic hepatitis in 73 cases (38.2%), hepatitis B in 71 cases (37.2%), hepatitis C in seven cases (3.7%), and other causes in 40 cases (21.0%). There were 41 patients with suspected SBP (21.5%), 18 patients with bacterascites (9.4%), and 132 patients who had no-AFI (69.1%). The demographic data are listed in **Table 1** and **Supplementary Table S1** and **Figure S1**.

**Table 1:** Clinical characteristics of enrolled patients.

	SBP (n = 41)	Bacterascites (n = 18)	no-AFI (n = 132)	*P^¶^*
Age (years, mean ± SD)	58.6 ± 8.3	59.4 ± 10.8	57.8 ± 9.2	0.766
Gender (male/female)	37/4	14/4	104/28	0.298
Etiology				
Alcohol	17 (41.5)	9 (50.0)	47 (35.6)	0.934
HBV	15 (37.5)	6 (33.3)	50 (37.9)	
HCV	1 (2.5)	0 (0)	6 (4.5)	
HBV plus alcohol	4 (9.8)	1 (5.6)	9 (6.8)	
Others	4 (9.8)	2 (11.1)	20 (15.2)	
Complications				
Ascites 2/3	24/17	10/8	80/52	0.906
Gastrointestinal bleeding	3 (7.5)	2 (11.1)	12 (9.0)	0.886
Hepatic encephalopathy	4 (10.0)	2 (11.1)	17 (12.9)	0.859
HRS	5 (12.5)	1 (5.6)	22 (16.7)	0.471
Laboratory parameters				
WBC (×10^9^/L), median (IQR)	7.6 (5.1, 12.0)	4.9 (3.2, 6.3)	3.7 (2.5, 6.3)	<0.01
neutrophil (%), median (IQR)	81.4 (70.6, 85.4)	73.9 (63.1, 86.7)	68.6 (59.2, 70.7)	<0.01
ALT (IU/L), median (IQR)	18.2 (8.1, 28.3)	17.4 (10.1, 24.3)	19.5 (11.7, 31.6)	0.297
AST(IU/L), median (IQR)	36.5 (18.7, 63.7)	24.2 (15.8, 49.8)	39.6 (24.9, 61.6)	0.147
PCT (ng/L), median (IQR)	0.18 (0.10, 1.84)	0.10 (0.05, 0.42)	0.15 (0.05, 1.82)	<0.01
CRP (mg/L), median (IQR)	17.4 (9.8, 48.8)	10.0 (7.2, 33.2)	10.0 (6.5, 31.9)	0.297
Albumin (g/dl), mean ± SD	30.2 ± 4.7	29.3 ± 4.4	29.7 ± 4.1	0.741
Total bilirubin (μmol/L), median (IQR)	54.8 (15.4, 168.5)	34.4 (21.3, 46.9)	52.3 (26.6, 99.8)	0.282
PTA, mean ± SD	65.7 ± 30.9	59.2 ± 16.8	58.7 ± 18.7	0.231
Serum creatinine (μmol/L), median (IQR)	91.2 (63.8, 179.5)	84.4 (64.8, 134.0)	81.7 (59.5, 120.3)	0.422
Platelets (10^9^/L), median (IQR)	91.5 (63.4, 141.3)	53.2 (26.2, 72.7)	79.5 (45.5, 114.8)	0.008
Ascites WBC count (×10^6^/L), median (IQR)	1,693 (765, 3620)	220 (92, 322)	200 (130, 620)	<0.01
Ascites PMN count (×10^6^/L), median (IQR)	1,437 (1,123, 3,587)	40 (8, 111)	54 (28, 94)	<0.01
Scores				
CTP	10.1 ± 1.8	9.6 ± 1.4	10.0 ± 1.6	0.688
MELD	16.0 ± 9.8	12.7 ± 5.3	14.2 ± 7.9	0.714

SD, standard deviation; IQR, interquartile range; HRS, hepatorenal syndrome; WBC, white blood cell; PCT, procalcitonin; CRP, C-reactive protein; ALT, alanine aminotransferase; AST, aspartate aminotransferase; PTA, prothrombin activity; CTP, Child-Turcotte-Pugh score; MELD, model for end-stage liver disease; PMN, polymorphonuclear neutrophils; AFI, ascitic fluid infection.

^¶^P-value from the Kruskal–Wallis test for continuous variables or the Fisher’s exact test for discrete variables that compares patients with SBP to patients with bacterascites and no-AFI.

### Analysis and valuation of ddPCR method

**Figure 2:**
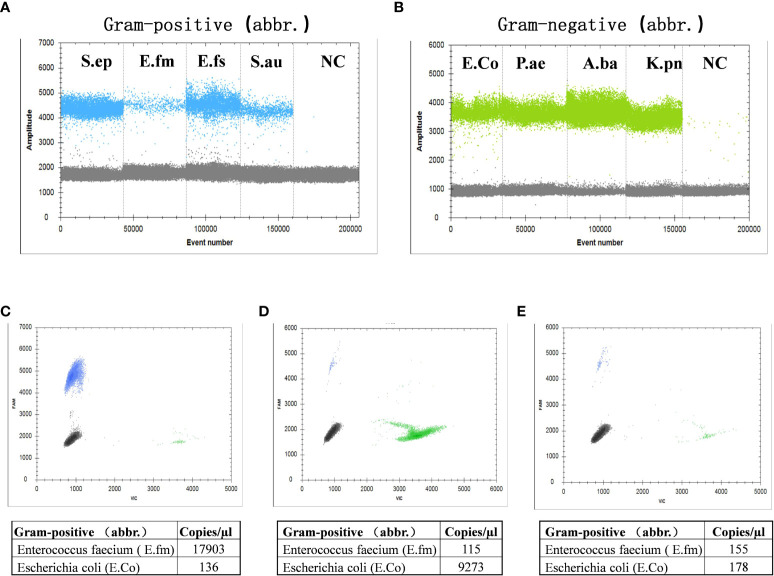
Detection of Gram-positive bacteria and Gram-negative bacteria using ddPCR. **(A)** Gram-positive bacteria, which included *Staphylococcus epidermidis*, *Enterococcus faecium*, *Enterococcus faecalis*, *Staphylococcus aureus*, and a negative control. **(B)** Gram-negative bacteria, which included *Escherichia coli*, *Pseudomonas aeruginosa*, *Acinetobacter baumannii*, *Klebsiella pneumoniae*, and a negative control. The interference test (different/mixed concentrations) of mixed *E. faecium* and *E. coli*; **(C)** 100:1; **(D)** 1:100; **(E)** 1:1.

Results showed that the clustering effect of ddPCR was best when the primer probe concentration was 400/200 nmol/L and the annealing temperature was 60°C (**Supplementary Table S2**, **Figure S2**). Representative Gram-positive and Gram-negative bacteria were selected for a specific probe test, which indicated that the probe could clearly distinguish Gram-positive or Gram-negative bacteria (**Figures 2A, B**). To determine whether the probe had cross interference in distinguishing Gram-positive and Gram-negative bacteria, we mixed *E. feacium* and *E. coli* in different concentrations (100:1, 1:100, and 1:1) and tested them. The results showed that the corresponding bactDNA loads were 4.3:2.1, 2.0:4.0, and 2.2:2.3 log copies/µl, indicating that reaction systems with different concentrations could be accurately classified and quantified (**Figures 2C–E**).

To determine the ddPCR detection limit, linearity, and repeatability, nine types of bacteria were spiked in a mixed system and subsequently detected by ddPCR. Serial dilutions of the above bacteria at known concentrations showed a good linearity (R^2^ = 0.97–0.99; **Supplementary Figure S3**), with three replicates at each dilution (1–5 log copies); the ddPCR detection limit was approximately 20–45 copies/µl for bacterial strains. Compared with traditional qPCR methods, the ddPCR showed better linearity and lower detection limits (**Supplementary Figure S4**).

### Clinical evaluation of an improved ddPCR quantitation method

**Figure 3:**
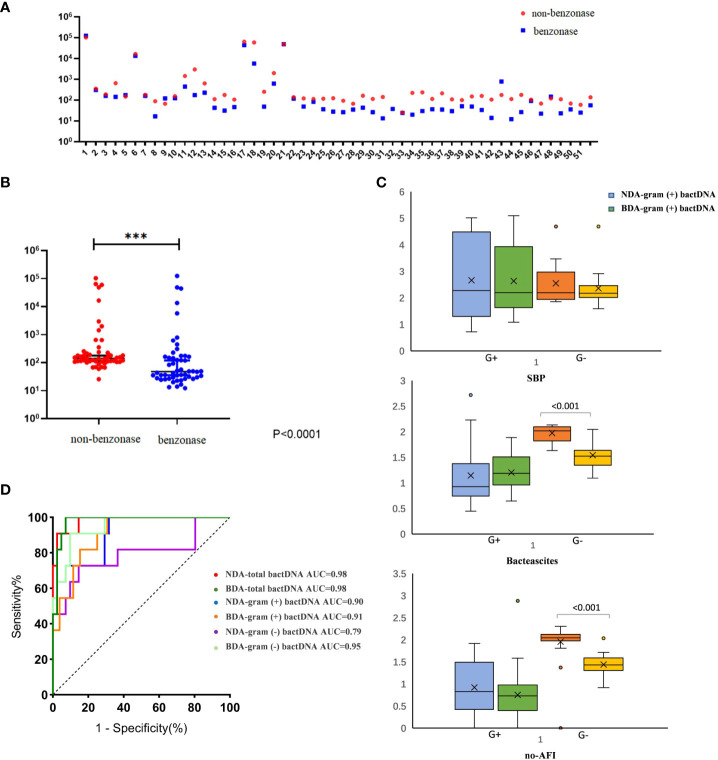
Clinical evaluation of an improved ddPCR quantitation method. **(A)** Fifty-four patients were selected in this comparison [SBP, 13; no-SBP, 41 (bacterascites, 15; no-AFI samples, 26)]. **(B)** Comparison between the non-dependent assay (NDA) and benzonase-dependent assay (BDA). **(C)** The results of Gram-positive and Gram-negative bactDNA quantitation of NDA and BDA in patients with SBP, bacteascites, and no-AFI. **(D)** ROC curves stratified by two different methods of NDA and BDA (n = 54 samples in total) based on clinical composite standards. NDA, non-dependent assay; BDA, benzonase-dependent assay; SBP, spontaneous bacterial peritonitis; AFI, ascites fluid infection; ***P < 0.001.

Benzonase endonuclease digestion was performed on the samples before detection; thus, all detected DNA came from live bacteria. We selected 54 cirrhotic ascites (13 SBP, 15 bacterascites, and 26 no-AFI samples) for validation. The results showed that, compared with the extraction method of none-dependent assay, the benzonase-dependent assay had a significant difference (P < 0.001) and even lower bactDNA load (1.75 vs. 1.5 copies/µl; **Figures 3A, B**); we found that this difference between the two extraction methods was mainly the extraction of Gram-negative bacteria from bacterascites and no-AFI (**Figure 3C**), which was conducive to distinguishing the interference of cell-free DNA fragments and the real bacterial infection with low loads. After the depletion of DNA fragments, the area under the ROC curve was as follows: bactDNA 0.98 (95% CI, 0.94–1.00), Gram-positive bactDNA 0.91 (95% CI, 0.84–0.99), and Gram-negative bactDNA 0.95 (95% CI, 0.88–1.00; **Figure 3D**), which indicated that DNA treatment with benzonase may have a better diagnostic value.

**Figure 4:**
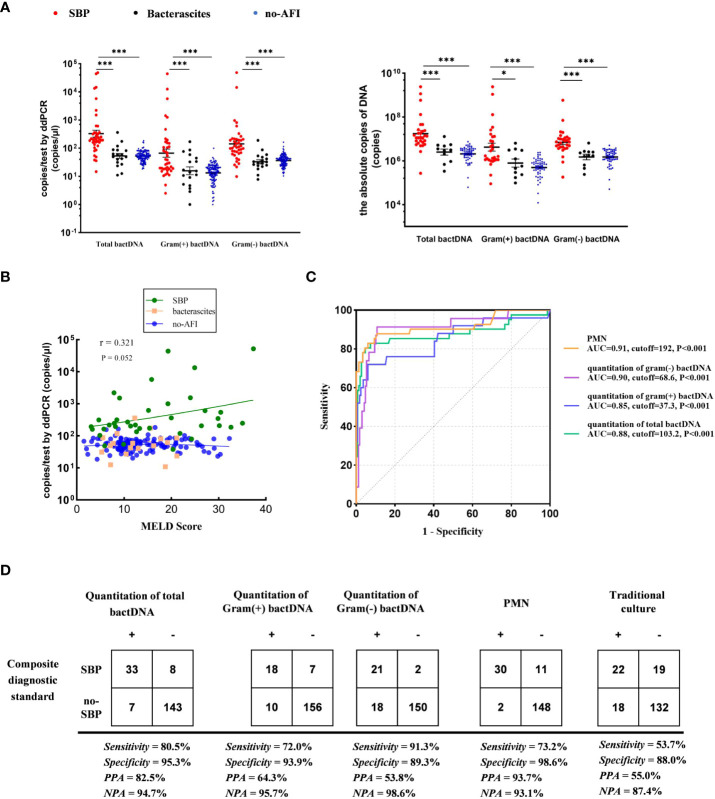
Accuracy of ddPCR testing in ascites. **(A)** Samples from 41 patients with SBP, 18 patients with bacterascites, and 132 patients with no-AFI. In the left panels, we compared the concentration of bactDNA. In the right panels, we obtained the absolute copies of bactDNA combined with ascitic volumes using virtual ultrasonography “three-point method”. **(B)** Correlation of bactDNA load with MELD. **(C)** ROC curves of bactDNA load from 191 samples based on clinical composite standards. **(D)** The 2 × 2 contingency tables for the validation of the quantification of bactDNA based on a clinical composite standard. MELD, Model for End-Stage Liver Disease; ROC, receiver operating characteristic; PPA, positive predictive agreement; NPA, negative predictive agreement; *P < 0.05, **P < 0.01, and ***P < 0.001.

We used the ultrasonic three-point method to evaluate and eliminate the influence of ascitic volumes and found that there was no significant variation in bactDNA levels between the concentration and the absolute copies of bactDNA combined with ascitic volumes (P > 0.53, **Figure 4A**). In addition, results showed that bactDNA loads in the ascites of patients with SBP (total of 2.8, Gram-positive of 1.9, and Gram-negative of 2.5 log copies/µl) were significantly higher than that of the patients with bacterascites (1.7, 1.3, and 1.5 log copies/µl) and the patients with no-AFI (2.0, 1.4, and 1.8 log copies/µl; P < 0.001; **Figure 4A**, **Supplementary Table S3**), whereas there was no significant difference between patients with bacterascites and ascites without infection. Then, we correlated the bactDNA load with the Model for End-Stage Liver Disease (MELD) score and found that bactDNA load in patients with SBP was weakly positively corelated with MELD (r = 0.321, P = 0.052; **Figure 4B**), whereas there was no correlation between bactDNA load and MELD in patients with bacterascites and ascites without infection.

### The enhanced sensitivity and specificity of bactDNA detection facilitates SBP diagnosis

Subsequently, we plotted ROC curves at varying bactDNA levels that corresponded to the SBP analysis. Results from 191 samples showed that the cutoff value of the bactDNA quantification was 103.2 copies/µl compared with the SBP diagnosis. For patients infected with Gram-positive and Gram-negative bacteria, the cutoff values were 37.3 and 68.6 copies/µl, respectively (**Figure 4C**).

At the optimal Youden index, which is derived from the ROC curve, the sensitivity and specificity of total bactDNA compared with the composite clinical standard were 80.5% (95% CI, 67.8%–93.2%) and 95.3% (95% CI, 91.1%–98.3%), respectively; the positive predictive agreement (PPA) and negative predictive agreement (NPA) were 82.5% and 94.7%, respectively. The diagnostic test revealed that 72% (95% CI, 53.1%–90.9%) sensitivity, 93.9% (95% CI, 90.3%–97.6%) specificity, 64.3% PPA, and 95.7% NPA of SBP were caused by Gram-positive bacteria; 91.3% (95% CI, 78.8%–100%) sensitivity, 89.3% (95% CI, 84.6%–94.0%) specificity, 53.8% PPA, and 98.6% NPA of SBP were caused by Gram-negative bacteria. In addition, the sensitivity and specificity of traditional culture compared with the composite clinical standard were 53.7% (95% CI, 40.0%–73.5%) and 88.0% (95% CI, 83.2%–93.4%), and PPA and NPA were 55.0% and 87.4% (**Figure 4D**), respectively. Therefore, as a novel tool, bactDNA based on ddPCR greatly improves microbial diagnosis in SBP.

### BactDNA levels as indicators of suspected infections in symptomatic patients with a PMN less than 250/mm^3^

**Figure 5:**
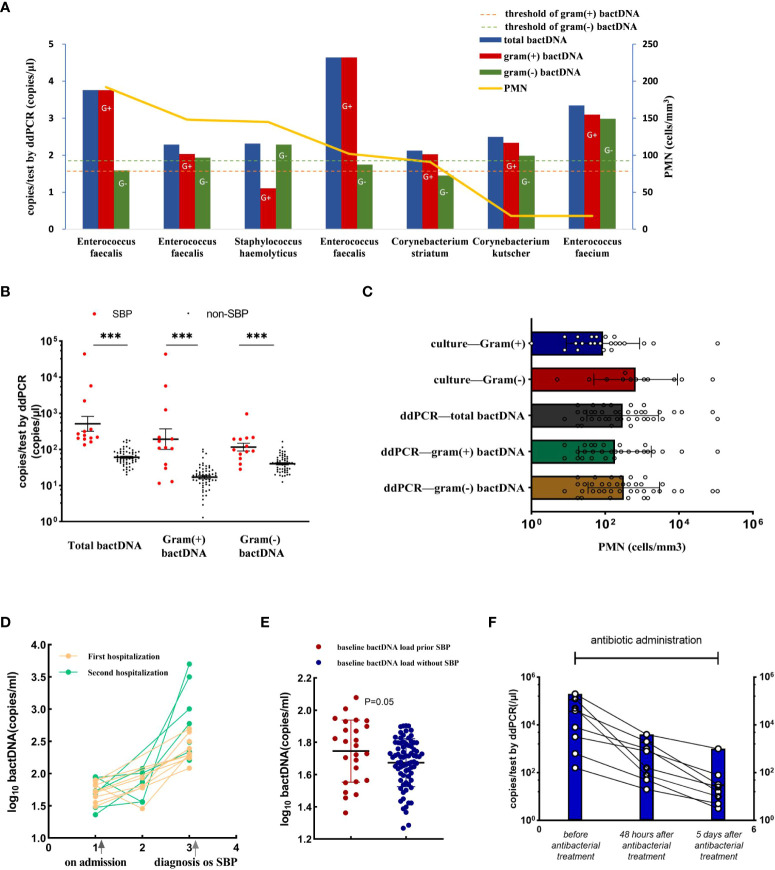
Accuracy of ddPCR testing in ascites and the bactDNA quantitation in patients with PMN < 250 cells/mm^3^. **(A)** The bactDNA load and PMN counts from seven patients diagnosed as SBP with PMN < 250/mm^3^. **(B)** Patients with PMN < 250/mm^3^ [SBP (n = 13) and no-SBP (n = 150)]. **(C)** PMN counts of different bacteria species in 191 samples based on culture and ddPCR. **(D)** Nine ascites without infection on admission developed SBP during hospitalization. Five patients were readmitted due to SBP diagnosis within 3 months, and five patients were readmitted due to SBP diagnosis within 3 months. **(E)** Baseline bactDNA load prior and without SBP development. **(F)** Dynamic changes of bactDNA load using ddPCR method in patients with SBP over time precede antibacterial treatment, 48 h and 5 days after antibacterial treatment (n = 8). PMN, polymorphonuclear; SBP, spontaneous bacterial peritonitis. ***P < 0.001.

The data showed that 163 of the 191 samples had PMN less than 250 cells/mm^3^, of which 13 patients were consulted by two infectious physicians and one hepatologist to consider SBP diagnosis and were treated with empirical antibiotics (see **Supplementary Table S4**). After treatment, the patients’ symptoms improved, and their diagnosis was clinically confirmed as SBP. Notably, among them, seven patients with culture positive were Gram-positive bacteria (**Figure 5A**), including *Enterococcus faecalis*, *Enterococcus faecium*, *Staphylococcus haemolyticus*, and *Corynebacterium*, which were consistent with a previous study in bacterascites (Li et al., 2020). In 13 patients with SBP with a PMN less than 250 cells/mm^3^, bactDNA amounts in ascites were significantly higher than those in patients with no-SBP (total of 2.7, Gram-positive of of 2.2, and Gram-negative of 2.1 log copies/µl vs. 1.7, 1.1, and 1.5 log copies/µl; P < 0.001; **Figure 5B**, **Supplementary Table S5**). According to the diagram of PMN counts, Gram-positive bacterial infections produce lower PMN counts than Gram-negative bacteria infection (**Figure 5C**). From our data, the cutoff value of PMN count was 192 cells/mm^3^ with 73.2% (95% CI, 59.4%–87.2%) sensitivity and 98.6% (95% CI, 97.3%–100%) specificity compared with composite diagnostic standard (**Figure 4D**). Therefore, we hypothesized that a PMN threshold of 250/mm^3^ was too high for the SBP diagnosis, especially for Gram-positive infections.

### BactDNA loads assist to predict the development of SBP and monitor antibiotic therapy in clinical practice

We selected 14 patients who developed SBP during hospitalization and collected available consecutive samples for three or more times, and nine ascites without infection on admission that developed SBP during hospitalization, with an average duration of 6 days. Five patients were readmitted due to SBP diagnosis within 3 months (**Figure 5D**). The results showed that the baseline bactDNA load was higher in patients with SBP development compared with patients without SBP development (55.7 copies/μl vs. 46.7 copies/μl; P = 0.05; **Figure 5E**), indicating that the bactDNA level was associated with incidence of SBP.

To assess dynamic change in bactDNA load before, during, and after SBP, eight patients with SBP (mean, 4 log copies/µl) were continuously observed before antibacterial treatment, 48 h and 5 days after antibacterial treatment. Results showed that the amount of bactDNA significantly decreased after 48 h of antimicrobial treatment (2.4 log copies/µl) and continued to decline with the use of antibiotics (1.4 log copies/µl; **Figure 5F**).

## Discussion

In this study, we described a rapid diagnostic method for bactDNA detection with low loads based on ddPCR to evaluate the bactDNA levels of abdominal infection in a large series of patients with cirrhosis and ascites. To our knowledge, this is the first study reporting detection of vital bacteria using benzonase to improve the SBP diagnosis. In particular, for symptomatic patients with PMN count <250/mm^3^, the bactDNA quantitation in ascites can be used to sensitively distinguish patients with suspected infections.

Benzonase has an advantage of removing free DNA fragments without affecting viable bacteria (Amar et al., 2021). Our data showed that, after the removal of free DNA, the concentration of bactDNA had generally decreased, whereas the area under the curve for diagnosing SBP was increased. However, we found that there was a significant decrease in gram-negative bacteria, which we speculated as either the increase of Gram-negative bactDNA from intestinal translocation to abdominal cavity or the increased destruction of Gram-negative bacteria due to the repeated freeze-thawing of samples during storage of ascitic samples. Notably, considering the effect of abdominal volume, we found that there was no significant variation of bactDNA between the concentration and the absolute copies of bactDNA combined with ascitic volume. Therefore, the abdominal volume dilution may not be a factor in affecting the bacterial absolute amount, which may be due to the high sensitivity of ddPCR detection technology.

Our study showed that the sensitivity and specificity of total bactDNA were 80.5% and 95.3% compared with the SBP diagnosis, which was consistent with the results of the bloodstream infection (Wouters et al., 2020). In addition, the bactDNA load is more likely to predict SBP development and reflect changes in the bacterial number with antibiotic use. The advantage of ddPCR assay is its culture independency and high sensitivity. It is a promising technology that not only performs absolute quantification but also accurately distinguishes Gram-positive and Gram-negative bacteria. Taken together, the bactDNA quantification in ascites by ddPCR is helpful for dynamically monitoring the changes of nucleic acid in pathogens and the effects of antibacterial treatment.

The SBP diagnosis is confirmed when the ascitic neutrophil count is ≥250 cells/mm^3^. However, in practice, some patients are symptomatic and have a PMN < 250 cells/mm^3^. Results from our data showed that those cases had a significant increase in bactDNA levels about 2–2.5 log copies/µl, indicating that, when PMN counts were not elevated, the bactDNA quantitation improved bacterial detection with low loads. In addition, we further observed that PMN count of 250/mm^3^ was probably higher with Gram-positive bacterial infections than with gram-negative bacterial infections. In the future, we will further study the relationship between PMN and the amounts of bacteria combined the bacteria species, thus further improving SBP’s diagnostic efficiency.

Some potential limitations of this study should be acknowledged. First, the presence of a small number of false-positive signals in the end data was found using the ddPCR method. Second, the bactDNA was not able to discriminate between bacterial ascites and ascites without infection in our study. The possible reasons are as follows:1) there were only 18 samples in patients with bacterascites, which may cause a statistical difference; 2) due to low positive culture rate of bacteria, there could also be low-load bacteria in non-infectious ascites that had not been detected by conventional method; and 3) because of the limitations of the laboratory environment, there was no significant difference in bactDNA load between the bacterascites and ascites without infection.

In addition, because of the detection time of 4 h and the cost of $5.20 per sample, the ddPCR method has an optimal applicational prospect in future clinical practice. Further prospective studies should be conducted to bactDNA levels on the guidance of clinical medication and its prognostic effect in patients with SBP to truly improve the SBP’s diagnostic value. Bacterascites may represent transient and spontaneously reversible colonization of ascites, or they may represent the first step in the development of SBP (European Association for the Study of the Liver., 2018). The bactDNA load in ascitic fluid by ddPCR may help identify the above two scenarios.

In conclusion, our study shows that the ascitic bactDNA quantitation by ddPCR is a promising approach that can be used to improve the SBP’s diagnostic accuracy, especially for patients who are symptomatic and have a PMN < 250 cells/mm^3^.

## Data availability statement

The original contributions presented in the study are included in the article/**Supplementary Material**. Further inquiries can be directed to the corresponding authors.

## Author contributions

HW, WH, and FW conceived the study. FW and ZH contributed to the study design. HW supervised all aspects of the study. WZ, ZW, and ZL were responsible for clinical data collection and verification. DC and SG were responsible for results collection and monitoring in the laboratory. HW and WH wrote the first draft of the manuscript. All authors critically reviewed the manuscript and contributed to writing-editing and approved the final version.

## Funding

This work was supported by the Beijing Institute of Hepatology Foundation (grant number Y-2021YS-1), the Clinical Research Special Fund of Wu Jieping Medical Foundation of China (grant number 320.6750.2022-08-1), and the Capital Health Research and Development of Special (grant number 2022-2-2183).

## Acknowledgments

The authors would like to thank Hui-Guo Ding and Pei-Zhi Li for their valuable input.

## Conflict of interest

The authors declare that the research was conducted in the absence of any commercial or financial relationships that could be construed as a potential conflict of interest.

## Publisher’s note

All claims expressed in this article are solely those of the authors and do not necessarily represent those of their affiliated organizations, or those of the publisher, the editors and the reviewers. Any product that may be evaluated in this article, or claim that may be made by its manufacturer, is not guaranteed or endorsed by the publisher.
