# Supplementary material for: Clinical evaluation of bacterial DNA using an improved droplet digital PCR for spontaneous bacterial peritonitis diagnosis
Source: Front Cell Infect Microbiol. 2022 Aug 18;12:876495. doi: 10.3389/fcimb.2022.876495 (PMC9433567; doi:10.3389/fcimb.2022.876495)
Supplement: Supplementary file 1 [file DataSheet_1.docx]

**Supplementary data**

*Original article*

**Clinical evaluation of bacterial DNA using an improved droplet digital PCR for spontaneous bacterial peritonitis diagnosis**

Hao-Xin Wu ^1^*, Wei Hou ^1^* , Wei Zhang ^1^ , Zheng Wang ^1^, Shan Guo^2^, De-Xi Chen^2^, Zhen Li ^1^ , Fei-Li Wei^2#^, Zhong-Jie Hu ^1#^

* Author Hao-Xin Wu and author Wei Hou are the co-first authors; ^#^Author Zhong-Jie Hu and author Fei-Li Wei are the co-corresponding authors.

**Affiliations**

^1^ Beijing Youan Hospital, Capital Medical University, Beijing, China.

^2^ Beijing Institute of Hepatology, Beijing YouAn Hospital, Capital Medical University, Beijing Precision Medicine and Transformation Engineering Technology Research Center of Hepatitis and Liver Cancer , Beijing, China.

**Key words:** peritonitis, bacterascites, diagnosis, quantitation, viable bacteria , ascitic volume, PMN, Benzonase dependent assay, gram-positive bacteria, gram-negative bacteria

**Corresponding author:** Dr. Zhong-Jie Hu (MD)

Beijing Youan Hospital, Capital Medical University, Beijing, China.

Phone number: 13501366613. Email:hzj6613@126.com.

**Alternate Corresponding author:** Dr. Fei-Li Wei

Beijing Institute of Hepatology, Beijing YouAn Hospital, Capital Medical University, Beijing Precision Medicine and Transformation Engineering Technology Research Center of Hepatitis and Liver Cancer , Beijing, China.

Phone number: 010-83997425. Email: [wflcn@126.com](mailto:wflcn@126.com).

**Contents**

**Inclusion and exclusion criteria**

**Supplementary Table S1.** Introduction of antibiotics in 191 patients with cirrhosis.

**Supplementary Table S2.** The verification conditions of primer and probe concentration

**Supplementary** **Table S3.** Comparison of bactDNA quantitation and PMN in three groups

**Supplementary** **Table S4.** The clinical characteristics of 13 patients with a PMN less than 250/mm^3^ .

**Supplementary** **Table S5.** Comparison of bactDNA quantitation and PMN in patients with a PMN less than 250 /mm^3^

**Supplementary Fig S1.** Comparison of bactDNA load between with and without antibiotic treatment.

**Supplementary Fig S2.** The ddPCR results of primer and probe concentration at 400/200 nM and annealing temperature at 60 ℃

**Supplementary Fig S3.** The results of repeatability and linearity.

**Supplementary Fig S4.** Comparison of linearity and limits of detection between ddPCR and qPCR method.

**Inclusion and exclusion criteria:**

Diagnostic evidence of spontaneous bacterial peritonitis: Whenever patients develop any of the following: 1) acute peritonitis: abdominal pain, abdominal tenderness or rebound tenderness, an increase in abdominal muscular tension, vomiting, and diarrhea or intestinal obstruction; 2) systemic inflammatory response syndrome: fever or normothermia, shivering, tachycardia, and tachypnea; 3) deterioration of liver function due to an unknown underlying cause; 4)hepatic encephalopathy; 5) shock; 6) refractory ascites, no response to diuretics, or renal failure; 7) acute gastrointestinal tract bleeding. And one or more of the following laboratory test abnormalities are present: 1) PMN ≥ 250 cells/mm^3^; 2) positive ascites bacteria culture; 3) procalcitonin (PCT) > 0.5 ng/mL, and infection of other sites is excluded. (EASL guidelines, J Hepatol 2018; Chinese guidelines, Hepatol Int 2019; AASLD Practice guidance, Hepatology 2021).

Inclusion criteria: Screening patients with cirrhosis (no etiology limitation) complicated with SBP, bacterascites and ascites without infection.

Exclusion criteria: acute HAV ,HEV ,EBV or CMV virus infection or infection of other sites. Ascites caused by other reasons, such as renal ascites, cardiac ascites, pregnancy drug abuse or HIV infection. Uncontrolled liver cancer or other systemic tumors. Previous organ transplant recipients receiving glucocorticoids or other immunosuppressive therapy. Rifaximin used in the past one month.

**Supplementary Table S1.** Introduction of antibiotics in 191 patients with cirrhosis.

|  | **SBP**  **(n=41)** | **Bacterascites**  **(n=18)** | **no-AFI**  **(n=132)** |
| --- | --- | --- | --- |
| Antibiotic treatment | 29 (70.2%) | 4 (22.2%) | 49 (37.1%) |
| Duration |  |  |  |
| <3 d | 14 (48.3%) | 0 (0.0%) | 12 (24.5%) |
| 3-7 d | 9 (31.0%) | 1 (25.0%) | 4 (8.2%) |
| >7 d | 6 (20.7%) | 3 (75.0%) | 33 (67.3%) |
| Empiric antibiotic therapy |  |  |  |
| Third-generation cephalosporins | 7 (24.1%) | 1 (25.0%) | 17 (34.7%) |
| Piperacillin/tazobactam ± linezolid | 6 (20.7%) | 2 (50.0%) | 12 (24.5%) |
| Meropenem ± linezolid | 8 (27.6%) | 1 (25.0%) | 9 (18.4%) |
| Other | 9 (31.0%) | 0 (0.0%) | 11 (22.4%) |

**Supplementary Table S2.** The verification conditions of primer and probe concentration.

| Primer | **Volume F（12um）** | **Volume R（12um）** | **Volume GRAM+（6um）** | **Volume GRAM-（6um）** | **water** | **Total volume** | **Final concentration** |
| --- | --- | --- | --- | --- | --- | --- | --- |
| 1 | 0.5 | 0.5 | 0.5 | 0.5 | 8 | 10 | 200/100 |
| 2 | 1 | 1 | 1 | 1 | 6 | 10 | 400/200 |
| 3 | 1.5 | 1.5 | 1.5 | 1.5 | 4 | 10 | 600/300 |
| 4 | 2 | 2 | 2 | 2 | 2 | 10 | 800/400 |

**Supplementary Table S3.** Comparison of bactDNA quantitation and PMN in three groups

|  | **SBP (n=41)** | **Bacterascites (n=18)** | **no-AFI (n=132)** | ***H*** | ***P*** |
| --- | --- | --- | --- | --- | --- |
| Quantitation of bactDNA (copies/µl) | 207.8  (140.7,361.5) | 58.6  (41.9,86.4) | 53.5  (40.3,73.5) | 60.861 | <0.001 |
| Gram-positive (copies/µl) | 34.1  (18.2,153.0) | 16.2  (10.3,48.1) | 15.0  (8.8,21.9) | 27.868 | <0.001 |
| Gram-negative (copies/µl) | 108.8  (65.8,207.2) | 32.9  (26.0,49.3) | 38.7  (25.8,48.6) | 50.945 | <0.001 |
| PMN (cells/mm^3^) | 427.5  (132.3,1132.0) | 47.5  (10.3,83.3) | 29.0  (17.0,54.5) | 52.987 | <0.001 |
| Total bactDNA*（copies） | 14.1E+06  (7.9,25.0) | 5.9E+06  (3.1,8.1) | 2.6E+06  (1.4,3.5) | 25.561 | <0.001 |
| Gram-positive | 6.4E+06  (1.1,16.2) | 14.4E+05  (5.7,35.1) | 6.0E+05  (3.3,9.2) | 19.281 | <0.001 |
| Gram-negative | 6.9E+06  (4.7,10.0) | 3.2E+06  (2.6,4.6) | 1.8E+06  (1.1,2.8) | 24.197 | <0.001 |

SBP: spontaneous bacterial peritonitis. AFI: ascitic fluid infection.

P value was conducted by Kruskal-Wallis test that compares patients with SBP to patients with bacterascites and no-AFI.

* Total bactDNA = concentration of bacterial DNA × ascitic volume. Virtual ultrasonography “Three-point method” was used to predict the ascitic volume，the formula is：ascitic volume (ml) = (1100/3)×(A+B+0.5×C)^[^[[1]](#endnote-1)^]^ . Virtual ultrasonography “Three-point method”：A: The thickness of the right subphrenic space was acquired at the maximal distance by placing the US probe vertically at the intercostal space(cm); B: The maximum thickness of the right paracolic was measured at the umbilical level(cm); C: The peri-bladder space was defined as the maximal distance between the abdominal wall and bladder by placing the US probe vertically (cm).

**Supplementary Table S4.** The clinical characteristics of 13 patients with PMN < 250/mm3 .

| **Patients (n=13)** | **PMN<250/mm^3^ with following symptom** |
| --- | --- |
| 6 | Obvious abdominal pain, abdominal tenderness, or rebound tenderness. |
| 1 | Abdominal tenderness and intestinal obstruction. |
| 2 | Fever with systemic inflammatory response syndrome and deterioration of the liver and kidney function. |
| 3 | hepatic encephalopathy without inducement. |
| 1 | no response to diuretics and had an acute kidney injury. |

**Supplementary Table S5.** Comparison bactDNA quantitation and PMN in patients with PMN ≤ 250 /mm3.

|  | **SBP (n=13)** | **no-SBP (n=150)** | ***U*** | ***P*** |
| --- | --- | --- | --- | --- |
| Quantitation of bactDNA (copies/µl) | 231.4(192.8,822.8) | 53.6(40.6,73.1) | -6.138 | <0.001 |
| Gram-positive (copies/µl) | 110.0(27.2,474.4) | 15.2(9.0,23.3) | -4.576 | <0.001 |
| Gram-negative (copies/µl) | 110.0(69.7,196.8) | 38.4(25.8,48.1) | -4.908 | <0.001 |
| PMN ( /mm^3^) | 86.5(18.0,145.8) | 29.0(16.0,54.0) | -2.287 | 0.022 |
| Total bactDNA (copies) | 1.6(1.0,6.3)E+07 | 2.7(1.5,3.6)E+06 | -3.912 | <0.001 |
| Gram-positive | 10.6(1.5,619.9)E+06 | 6.0(3.6,9.8)E+05 | -3.586 | <0.001 |
| Gram-negative | 5.9(4.5,12.4)E+06 | 1.8(1.1,2.9)E+06 | -3.611 | <0.001 |

**Supplementary Fig S1.**Comparison of bactDNA load between with and without antibiotic treatment. The results of three groups showed that bactDNA load was slightly higher before than after antibiotic treatment, but there was no statistical difference between the two cohorts.


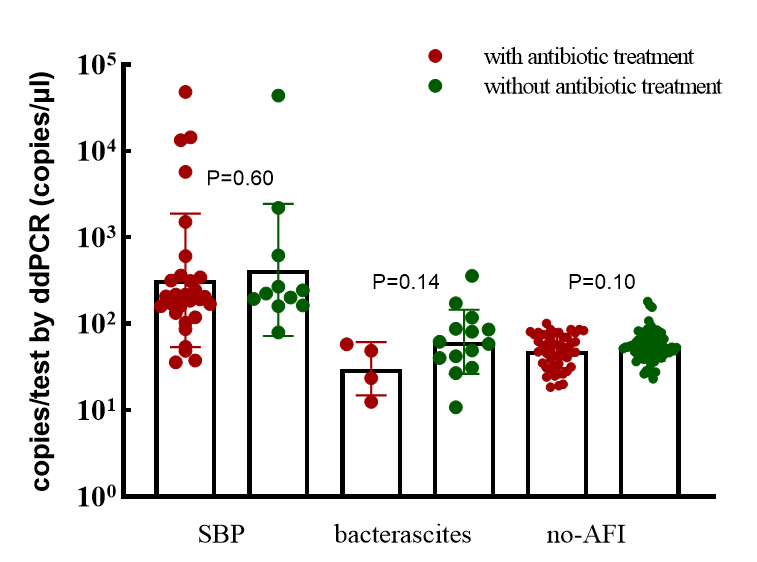


**
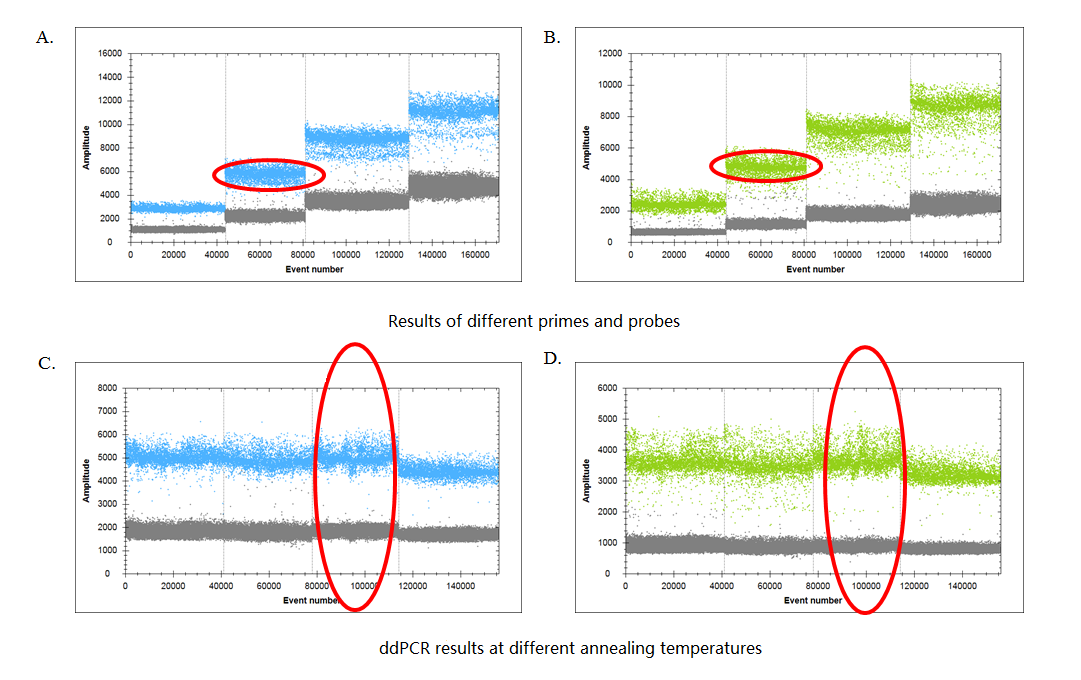
Supplementary Fig S2.** The ddPCR results of primer and probe concentration at 400/200 nM and annealing temperature at 60℃

**Supplementary Fig S3.** Repeatability and linearity results (R2 = 0.97- 0.99)

**Supplementary Fig S4.** Comparison of linearity and limits of detection between ddPCR and qPCR method.

**
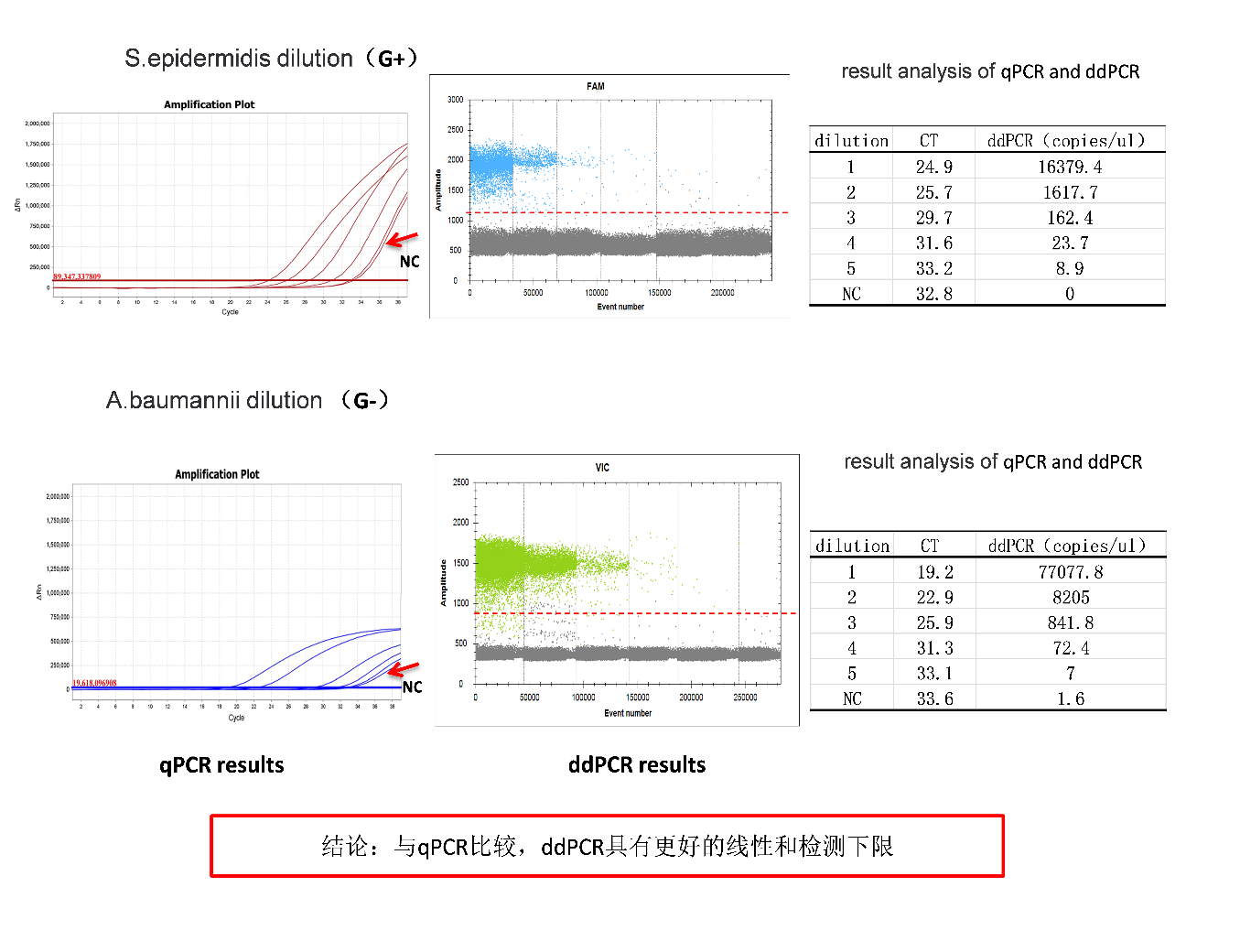
**

1. [] Hirooka M , Koizumi Y , Imai Y , et al. Validation trial for efficacy of ultrasonographic measurement method to predict ascitic volume using virtual ultrasonography[J]. Journal of Medical Ultrasonics, 2018. [↑](#endnote-ref-1)
